# CloudSEN12+: The largest dataset of expert-labeled pixels for cloud and cloud shadow detection in Sentinel-2

**DOI:** 10.1016/j.dib.2024.110852

**Published:** 2024-08-19

**Authors:** Cesar Aybar, Lesly Bautista, David Montero, Julio Contreras, Daryl Ayala, Fernando Prudencio, Jhomira Loja, Luis Ysuhuaylas, Fernando Herrera, Karen Gonzales, Jeanett Valladares, Lucy A. Flores, Evelin Mamani, Maria Quiñonez, Rai Fajardo, Wendy Espinoza, Antonio Limas, Roy Yali, Alejandro Alcántara, Martin Leyva, Raúl Loayza-Muro, Bram Willems, Gonzalo Mateo-García, Luis Gómez-Chova

**Affiliations:** a Image Processing Laboratory, University of Valencia, 46980 Valencia, Spain; b High Mountain Ecosystem Research Group, National University of San Marcos, 15081 Lima, Peru; c Centro de Competencias del Agua (CCA), Lima, Peru; d Remote Sensing Centre for Earth System Research (RSC4Earth), Leipzig University, 04103 Leipzig, Germany; e Universidad Nacional Agraria La Molina, 15024 Lima, Peru; f Universidad Nacional Federico Villarreal, 15082 Lima, Peru; g Universidad Nacional Santiago Antúnez de Mayolo, 02002 Huaraz, Peru; h Universidad Nacional Agraria de la Selva, 10131 Tingo María, Peru; i Department of Cartographic and Land Engineering, Universidad de Salamanca, 05003 Ávila, Spain; j Laboratory of Ecotoxicology, Faculty of Sciences and Philosophy, Universidad Peruana Cayetano Heredia, 15102 Lima, Peru; k German Centre for Integrative Biodiversity Research (iDiv) Halle-Jena-Leipzig, 04103 Leipzig, Germany

**Keywords:** Sentinel-2, Thin cloud, Cloud shadow, IRIS, U-net, Global dataset

## Abstract

Detecting and screening clouds is the first step in most optical remote sensing analyses. Cloud formation is diverse, presenting many shapes, thicknesses, and altitudes. This variety poses a significant challenge to the development of effective cloud detection algorithms, as most datasets lack an unbiased representation. To address this issue, we have built CloudSEN12+, a significant expansion of the CloudSEN12 dataset. This new dataset doubles the expert-labeled annotations, making it the largest cloud and cloud shadow detection dataset for Sentinel-2 imagery up to date. We have carefully reviewed and refined our previous annotations to ensure maximum trustworthiness. We expect CloudSEN12+ will be a valuable resource for the cloud detection research community.

Specifications Table

**Table:** 

Subject	Computers in Earth Sciences.
Specific subject area	Cloud detection in optical remote sensing data.
Type of data	GeoTIFF imagery CSV Table
Data collection	The dataset comprises Sentinel-2 Level 1C (S2) imagery associated with hand-crafted labels. The S2 images are retrieved from Google Earth Engine [9] using the R client interface [3]. Sixteen experts generated the labels following a strict cloud detection protocol. Additionally, we have incorporated cloud detection predictions generated by the CloudSEN12 UnetMobV2 model [1] into each image.
Data source location	50,249 Sentinel-2 L1C image patches distributed around all the continents except Antarctica. This represents a total extent of 1,283,256 km². Primary data:https://developers.google.com/earth-engine/datasets/catalog/COPERNICUS_S2_HARMONIZED
Data accessibility	Repository name: Science Data Bank Data identification number: 10.57760/sciencedb.17702 Direct URL to data: https://www.scidb.cn/en/detail?dataSetId=2036f4657b094edfbb099053d6024b08
Related research article	Aybar, C., Ysuhuaylas, L., Loja, J. et al. CloudSEN12, a global dataset for semantic understanding of cloud and cloud shadow in Sentinel-2. Sci Data 9, 782 (2022). https://doi.org/10.1038/s41597-022-01878-2

## 1 Value of the Data

**Fig. 1:**
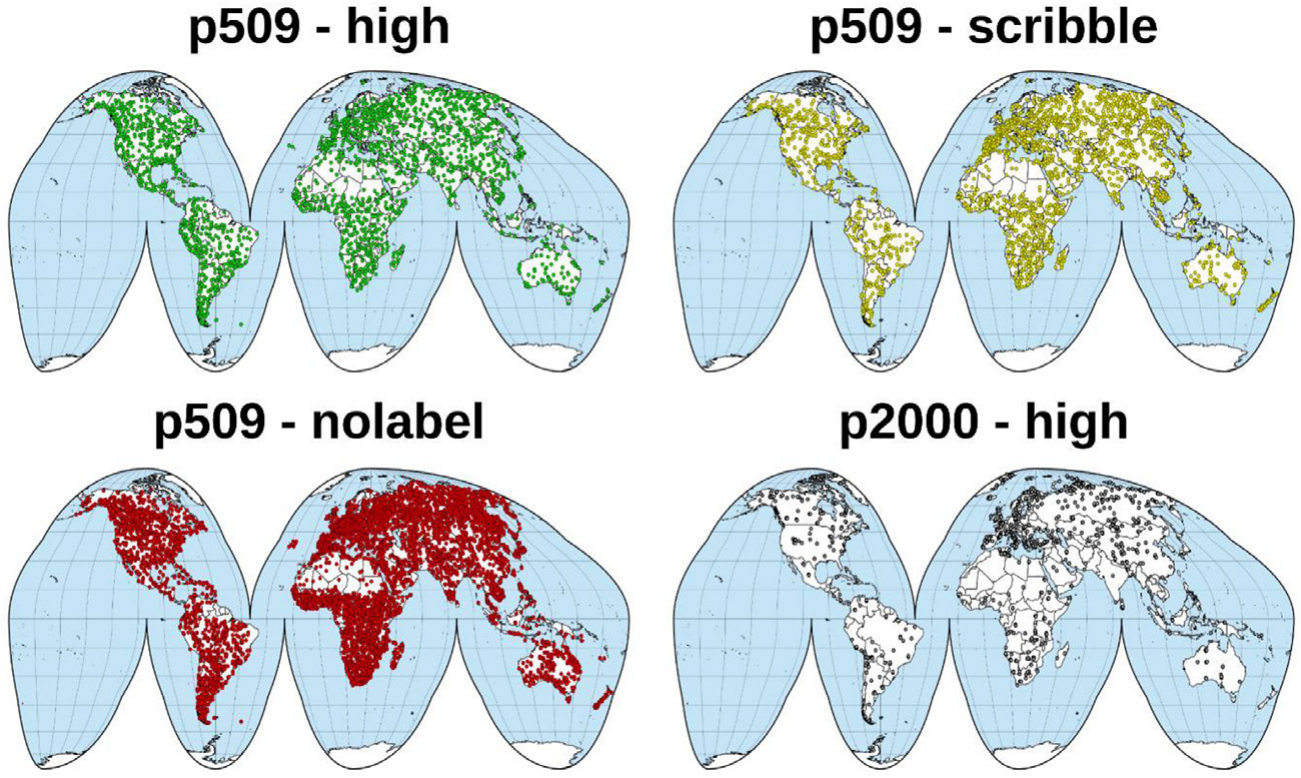
CloudSEN12+ spatial coverage. The terms p509 and p2000 denote the patch size 509 × 509 and 2000 × 2000, respectively. ‘high’, ‘scribble’, and ‘nolabel’ refer to the types of expert-labeled annotations.
• It includes images from various regions worldwide, providing a geographically diverse dataset that can help improving the generalization of trained cloud detection algorithms.
• It provides high-quality expert-labeled annotations using a consistent and well-defined labeling protocol in two patch sizes: 509×509 and 2000×2000 10 m pixels. As part of the legacy of version 1 (CloudSEN12 dataset), it also provides scribble expert-labeled annotations and no-label patches.
• It can serve as a foundation for other remote sensing (RS) sensors, enabling researchers to transfer the knowledge gained from S2 to similar sensors, such as Landsat or multiple of small-size RGBNIR optical satellites.
• This dataset is licensed under CC0, which puts it in the public domain and allows anyone to use, modify, and distribute it without permission or attribution.

• The collection consists of more than 50,000 S2 image patches (Fig. 1). It covers diverse cloud scenes with varying shapes, thicknesses, sizes, and altitudes, providing a comprehensive dataset for training and testing cloud detection algorithms.

## 2 Background

**Fig. 2:**
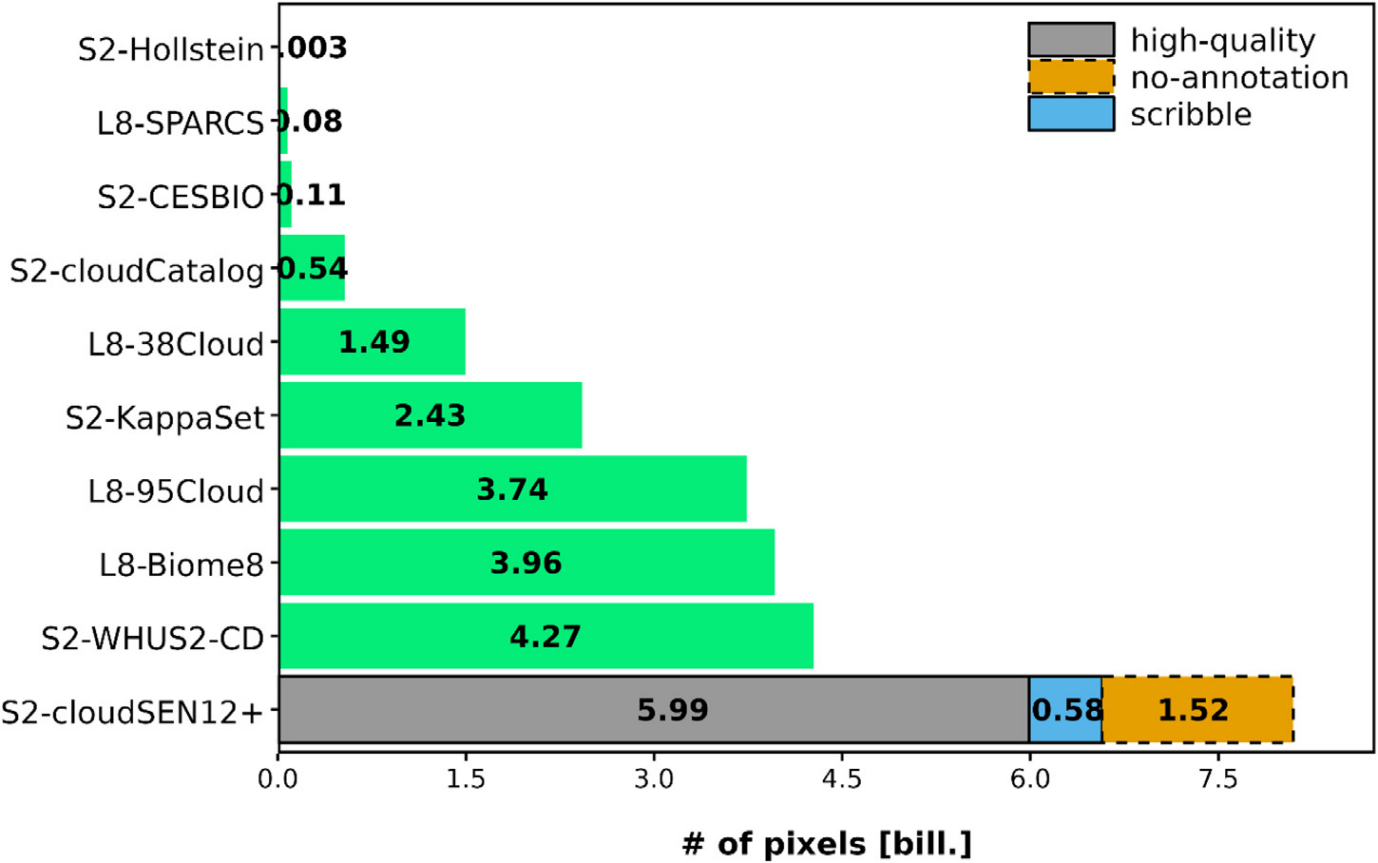
Comparison of CloudSEN12+ with other Landsat and S2 cloud detection datasets.

Accurately detecting clouds in optical RS imagery is critical for various environmental and Earth observation studies [5,10,13]. Clouds obstruct and contaminate surface reflectance signal, causing inaccuracies when retrieving land and ocean parameters [15]. To tackle this challenge, there has been a growing interest in creating robust algorithms for cloud screening from RS imagery. From a data-driven perspective, the first step in developing cloud detection algorithms is to create a training dataset. Several datasets (Fig. 2) have been created for this purpose [4,8,6,11]. However, they have limitations and particularly they lack diversity across cloud types and geographies [14]. The CloudSEN12 dataset [1] was designed to address these issues, nevertheless models trained in CloudSEN12 are still not exempt of errors [2]. The novel CloudSEN12+ tackles these errors by extending CloudSEN12 with more labels in challenging areas, increasing the size of the patches to improve shadow detection, and curating several of the original labels following extra quality control procedures. With these improvements, we expect to push forward the accuracy of cloud detection models.

## 3 Data Description

**Fig. 3:**
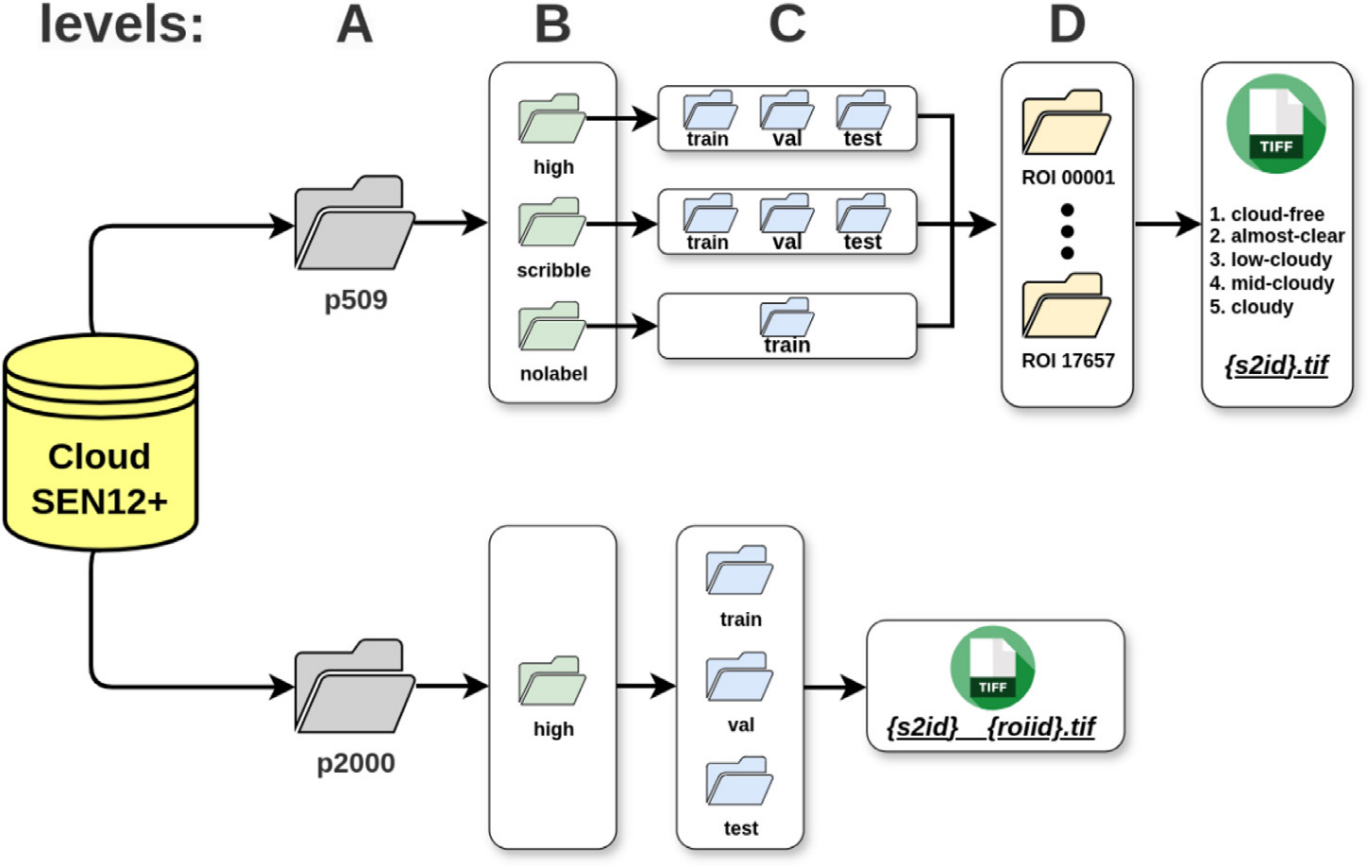
The CloudSEN12+ dataset is structured hierarchically, with the top level (A) dividing the dataset into two main categories: *p509* and *p2000* image patches, represented by gray folders. Moving to the next level (B), the images are further organized based on the label type, with each label type having a different folder. Within each label type, an additional level (C) groups the images based on a block of random data splitting, represented by blue folders. Moreover, within the *p509* category, there is an additional division based on geographic location, highlighted by yellow folders (D). Each yellow folder contains a set of five distinct images with cloud cover ranging from 0 % (cloud-free) to near 100% (cloudy).

Table 1 presents the number of image patches in each subfolder. The dataset is divided into two main collections, p509 and p2000, as shown in Fig. 3A. These numbers correspond to the image patch sizes of 509×509 and 2000×2000 pixels, respectively.

**Table 1:** Summary of image patch distribution across CloudSEN12+ subfolders.

patch size	label type	train	val	test
p509	High	8490	535	975
	Scribble	8785	560	655
	Nolabel	29400	0	0
p2000	High	687	77	85

The initial p509 folder is further divided into three groups depending on the manual label type: ‘high’, ‘scribble’, and ‘nolabel’ (Fig. 3B). S2 images labeled as ‘high’ indicate that each pixel within the image (i.e., 509×509) is associated with a cloud semantic category described in Table 2. This subset is ideal for training machine learning models since they require pixel-level accuracy to learn complex patterns and distinctions in cloud formations. Using the Intelligently Reinforced Image Segmentation (IRIS) [12] brush tool, S2 images within the ‘scribble’ subset cover only a small percentage of pixels with annotations (less than 5%). These labels are particularly useful for validation, offering a balanced representation of pixels far and near to edges —areas where cloud detection algorithms commonly fail [1]. Finally, S2 images in the ‘nolabel’ subset do not have human annotations. However, we include in all patches the accurate cloud masks generated by the CloudSEN12 UnetMobV2 model, which can serve as a basis for training a cloud detection model before performing a fine-tuning with the ‘high’ quality human labels. Both the ‘high’ and ‘scribble’ categories are segmented into three subfolders (train, val, and test), while ‘nolabel’ only contains the train subfolder (Fig. 3C).

**Table 2:** Cloud semantic categories considered in CloudSEN12+. Lower priority levels indicate greater relevance. Some classes have a greater impact on the overall quality of the image. To measure this impact fairly, we have introduced a 'Priority' column to indicate the classes that require greater attention from labelers. Lower priority levels indicate higher relevance.

Code	Class	Description	Priority
0	Clear	Pixels without cloud and cloud shadow contamination. They are primarily identified using bands B4- B3-B2, B1-B12-B13, and the cirrus band.	4
1	Thick Cloud	Opaque clouds that block all the reflected light from the Earth's surface. We identify them by assuming clouds exhibit distinctive shapes and maintain higher reflectance values in bands B4-B3-B2, B1-B12-B13, and the cirrus band.	1
2	Thin Cloud	Semitransparent clouds that alter the surface spectral signal but still allow to recognize the background. This is the hardest class to identify. We utilize CloudApp [1] to better understand the background, both with and without cloud cover.	3
3	Cloud Shadow	Dark pixels where light is occluded by thick or thin clouds. Cloud shadows depend on clouds presence and, by considering the solar position, we can identify and map these shadows through a reasoned projection of the cloud shape.	2

The final level (Fig. 3D) represents the geographic diversity, with each ROI illustrating a distinct area. Within each ROI, there are five images categorized by different levels (%) of cloud coverage: cloud-free (0%), almost-clear (0–25%), low-cloudy (25–65%), mid-cloudy (45–65%), and cloudy (>65%).

The p2000 collection exclusively contains ‘high’ quality human annotations and is systematically organized into the train, val, and test subfolders. In both cases, p509 and p2000, the human annotators had the option to generate the labels with the initial support of a machine learning model assistant (see section Cloud detection protocol). In contrast to p509, the p2000 subset includes only one image per location. The p2000 collection is designed to enhance the performance of models initially trained on the p509 dataset by leveraging larger image patches. The models trained in p2000 patches should better capture the spatial autocorrelation between cloud and cloud shadow classes thanks to the wider receptive field.

Each image patch in *p509* and *p2000* comprises fifteen bands: thirteen corresponding to S2, one for the manual label (filled with NA for no label subset), and one for the automatic labels generated by CloudSEN12 UnetMobV2.

## 4 Experimental Design, Materials and Methods

CloudSEN12+ is an extension of the CloudSEN12 dataset which adds a set of new manually labeled images with a larger patch size (see subsection Large patch size collection) and improves the labels of 452 images identified by a label quality protocol (see subsection Semi-automatic label quality).

### 4.1 Sentinel-2 data

The S2 mission currently comprises two nearly identical satellites, Sentinel-2A and Sentinel-2B, launched in June 2015 and March 2017, respectively. These satellite products offer estimates of reflectance values across 13 spectral channels, covering the entire globe every five days [7]. The S2 imagery is freely distributed under an open data policy. In the CloudSEN12+ dataset, we use the L1C products that provide Top-of-Atmosphere surface reflectance. All image bands at 20m and 60m are unsampled to 10-meter resolution using nearest neighbor interpolation to have a uniform resolution across bands.

### 4.2 Large patch size collection

One of the most significant challenges in cloud semantic segmentation is accurately identifying cloud shadows [16]. Neural network models often struggle to differentiate between cloud shadows and other types of shadows, such as those originating from terrain or other objects. To address this problem, larger patches were added to make it easier for the networks to learn the spatial relationship between clouds and their shadows. Our selection process involved manually choosing images with high potential error for cloud shadows (see section Semi-automatic label quality). Furthermore, more bright regions like deserts and snow were included. Ultimately, 849 images were labeled. The final spatial distribution of the dataset can be seen in Fig. 1.

### 4.3 Cloud detection protocol

Creating human-generated labels for cloud detection can be a complex task, and several factors can contribute to potential inaccuracies. Firstly, defining borders between clear and cloud-contaminated areas is challenging, as individual priors and biases influence the thresholds and decisions used to differentiate them. Secondly, cloud detection is an imbalanced problem. Opaque and oval-shaped clouds are more commonly observed and labeled, which can result in the under-representation of less frequent cloud types, such as semi-transparent and elongated clouds. Third, semantic classes are not always mutually exclusive. Pixels within an image can simultaneously belong to multiple classes. For instance, a semi-transparent cirrus cloud may overlap an opaque cumulus cloud or a cloud shadow, creating mixed pixels. Finally, some classes have interdependence, as cloud shadows are inherently dependent on the existence of clouds.

**Fig. 4:**
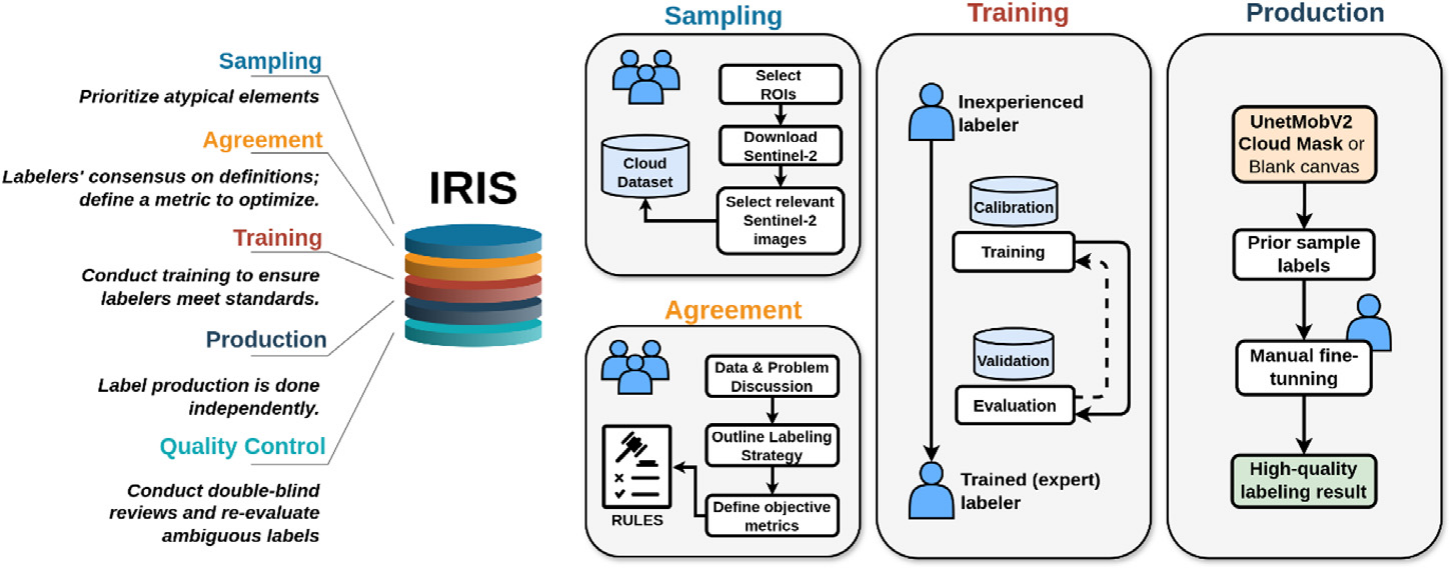
The image illustrates our cloud detection protocol, structured into five stages: Sampling, Agreement, Training, Production, and Quality Control. The IRIS graphical user interface is integral to each of these stages. The Quality Control section is detailed in Fig. 6.

**Fig. 5:**
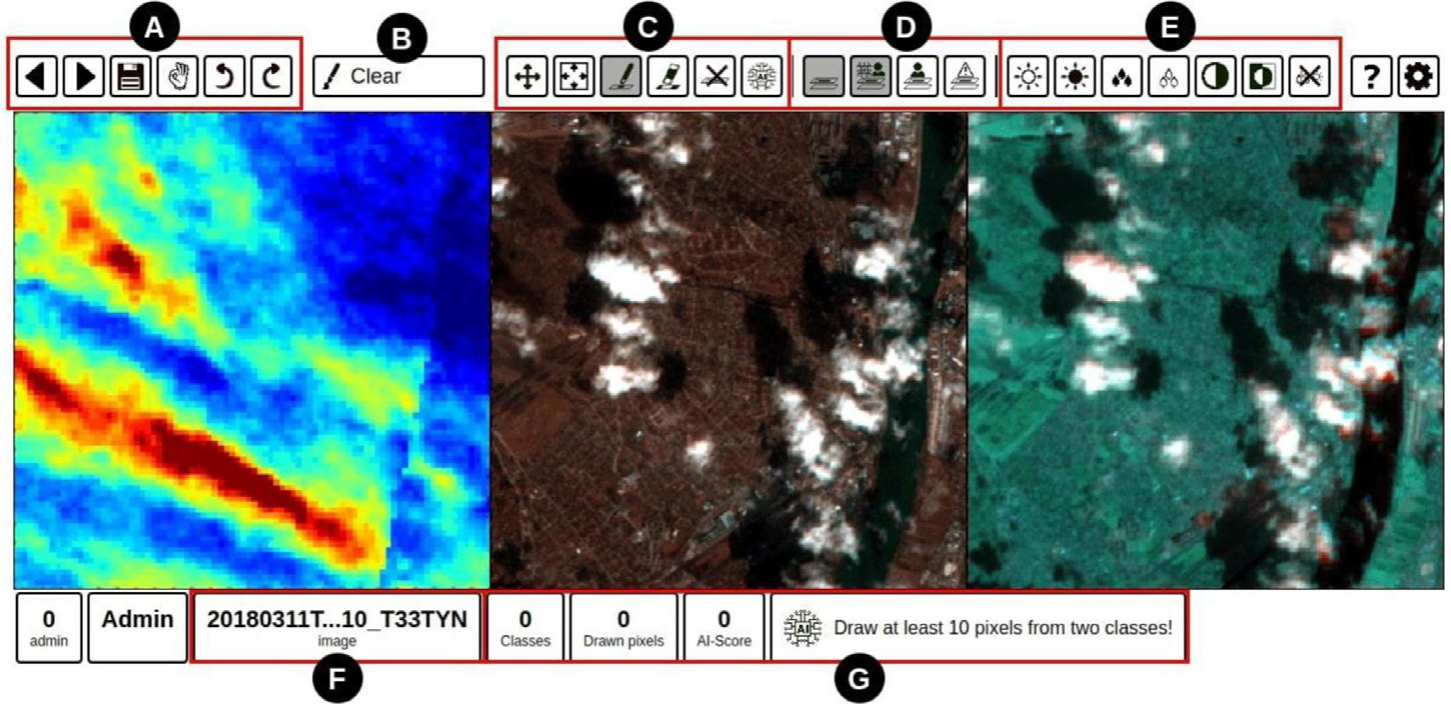
The IRIS (Intelligently Reinforced Image Segmentation) graphical user interface includes seven toolbars: A) Edit and navigation bar; B) Selection tool for drawing semantic classes; C) Drawing toolbar, with a final button to execute the GBDT algorithm that completes the mask using previous manual annotations; D) Testing toolbar, allowing comparison between human and AI annotations; E) Image contrast toolbar, which adjusts brightness and saturation; F) Image metadata section, displaying a thumbnail and IP location via Google Maps; G) Machine learning summary support, showing GBDT performance metrics. The IRIS interface includes views of the Cirrus band, Red-Green-Blue, and Blue-SWIR1-SWIR2 bands by default. This image corresponds to the one found in the supplementary information of [1].

Recognizing and accurately labeling the complex cloud patterns requires specialized knowledge. To achieve the highest label accuracy for CloudSEN12+, we have meticulously designed a comprehensive five-step protocol that effectively addresses the unique challenges that cloud labeling poses (Fig. 4). This protocol is not only applicable to CloudSEN12+ but can also be adapted to enhance labeling accuracy in various remote sensing tasks. Our protocol is built around IRIS, a semi-automatic tool designed for manual segmentation of multi-spectral and geospatial imagery (Fig. 5). This tool aids in achieving precise and consistent labeling by leveraging machine learning assistance while allowing for human oversight and adjustment.

1. **Sampling**: Manual sampling remains the most effective approach, despite being time-consuming. The vast array of cloud types and their unique characteristics demand careful consideration. To address this, labelers prioritize atypical clouds, such as contrails, ice clouds, and haze/fog, over more common varieties like cumulus and stratus. Furthermore, using a reference model to determine which data points should be included in a dataset is helpful. Labelers make informed decisions about which samples to prioritize by comparing human interpretation with the reference model.
2. **Agreement**: Before starting the labeling process, all labelers come to a mutual agreement on the definitions and criteria for each semantic class, creating common guidelines (refer to Table 2). When ambiguity arises, and a pixel could belong to multiple classes, the priority attribute is established to determine the final allocation based on the higher priority class. This prioritization strategy ensures consistent labeling decisions, particularly in borderline cases. Additionally, all labelers agree on a specific metric to optimize. For CloudSEN12+, the chosen metric is the F2-score, which places more emphasis on recall in the evaluation. This prioritization highlights errors in thick clouds and cloud shadows over those in thin clouds and clear classes. Lastly, specific band combinations are established to aid cloud detection (refer to Table 2).
3. **Training**: Labelers follow a comprehensive training program designed to teach them to agree with the labeling software and enhance their skills in ambiguous scenarios. The training begins with an in- depth review of accurately labeled image examples, enabling participants to align with the established standards and expectations for labeling. Furthermore, the training encompasses hands-on practical sessions, during which labelers put their learning in real-time scenarios and receive constructive feed- back to sharpen their labeling skills. The training stage is pivotal in ensuring that all contributors are thoroughly prepared and maintain consistency in their labeling efforts.
4. **Production**: The labeling process is conducted in this stage. Labelers can start labeling from a blank canvas or fine-tune the preliminary cloud mask predictions provided by the CloudSEN12 UnetMobV2 model. Each labeler undertakes the task independently.
5. **Quality Control**: The labels go through a double-blind quality control process that involves all the labelers to ensure their integrity and accuracy. If more than two independent reviewers report a label, it is sent back to the production stage. Additionally, all human-generated labels exhibiting a *P_error_* equal to 1 (see *Semi-automatic label quality* section) are subject to a meticulous re-examination.

### 4.4 Semi-automatic label quality

**Fig. 6:**
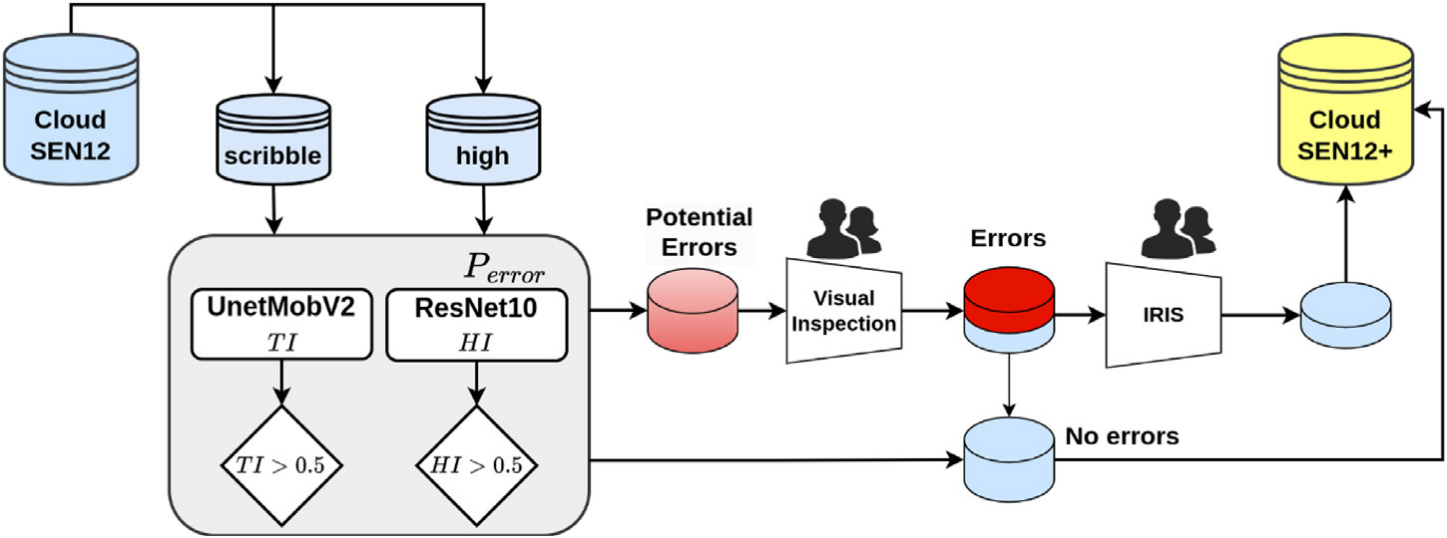
A high-level summary of our workflow to detect human errors. Prediction accuracy (TI) and sample difficulty (HI) are used to identify errors in high-quality and scribble subsets.

CloudSEN12+ employs a dual-scoring approach to detect potential human errors in semantic segmentation [2]. The methodology is illustrated in Fig. 6. Initially, we calculate the trustworthiness index (TI), which compares the cloud mask prediction from a reference model with the corresponding human annotations used as ground truth. We have selected the CloudSEN12 UnetMobV2 as the best available reference model. The TI is computed using the F2 multi-class score, adopting a one-vs-all macro strategy:

$$\mathrm{TI}=\;\frac{1}{C}\sum_{c=1}^{C}\frac{TP_{c}}{TP_{c}+0.2FP_{c}+0.8FN_{c}}$$

Where, *FN* represents false negatives, *FP* false positives and *TP* true positives; c identifies each class (clear, thin cloud, thick cloud and cloud shadow), and C the number of classes (C = 4).

Annotation errors are more susceptible in challenging scenarios, such as class boundaries, intricate cloud shapes, or insufficient contextual information. To address this, we incorporate a Hardness Index (*HI)* that considers the perceived difficulty of the labelers during the annotation process. In order to build this index, a ResNet-10 model is trained with the S2 images as input and the labelers’ perceived difficulty as the target, which is included in the metadata of the CloudSEN12 dataset [1]. This model effectively accounts for the complexity of the annotation task and helps identify areas where errors are more likely to occur.

The *TI* and *HI* indices are estimated for all the image patches in CloudSEN12+. The potential errors *P_error_* are detected by considering a simple combination of these indices:

$$P_{\mathrm{error}}=\;\left\{ \begin{array}{cc} 1 & \mathrm{if}\;\mathrm{TI}<0.3\;\mathrm{and}\;\mathrm{HI}>0.5\\ 0 & \mathrm{Otherwise} \end{array}\right.$$

**Fig. 7:**
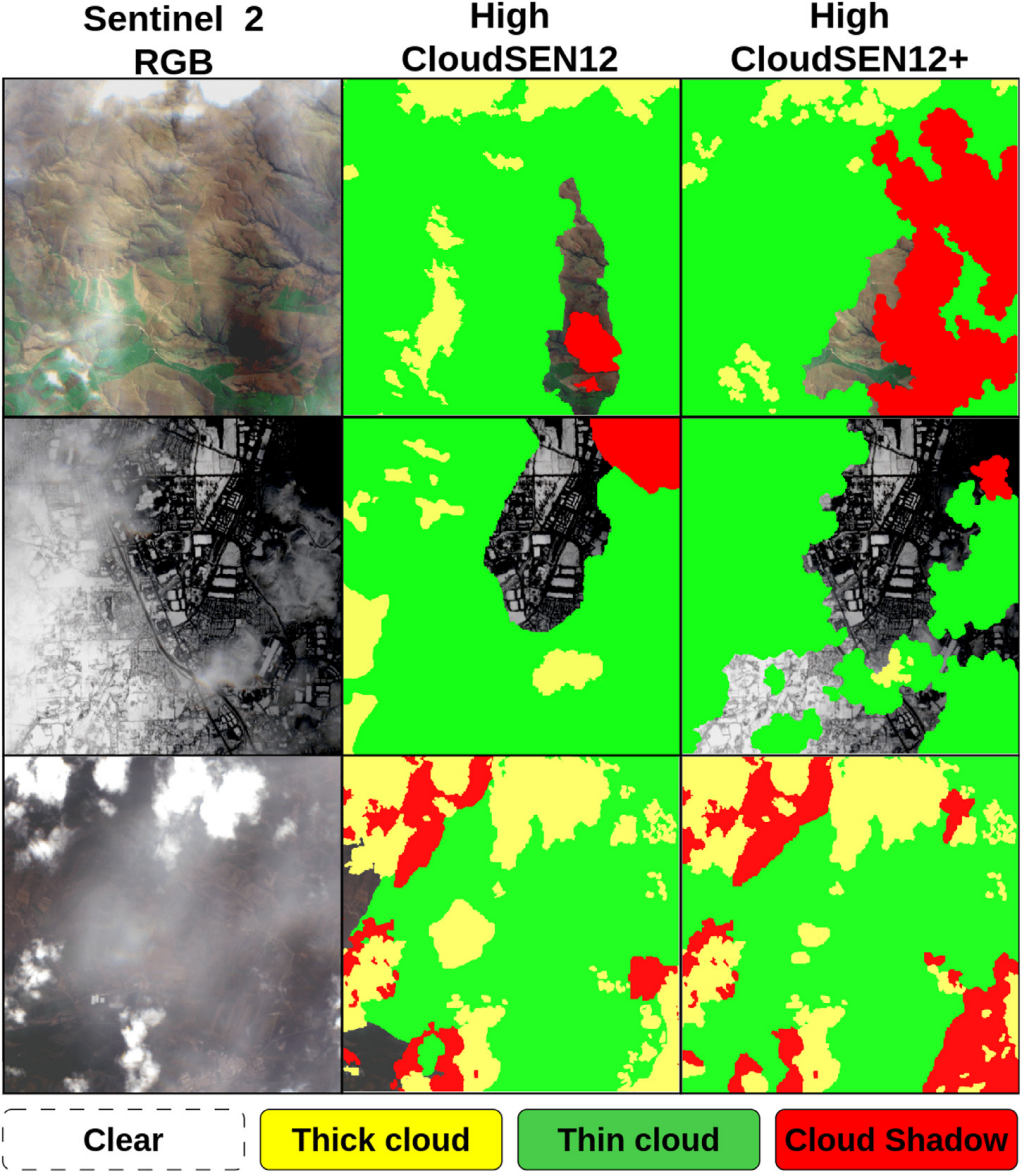
Label correction in the ‘high’ quality subset. The images come from the ROIs: 10133, 720, and 1953.

**Fig. 8:**
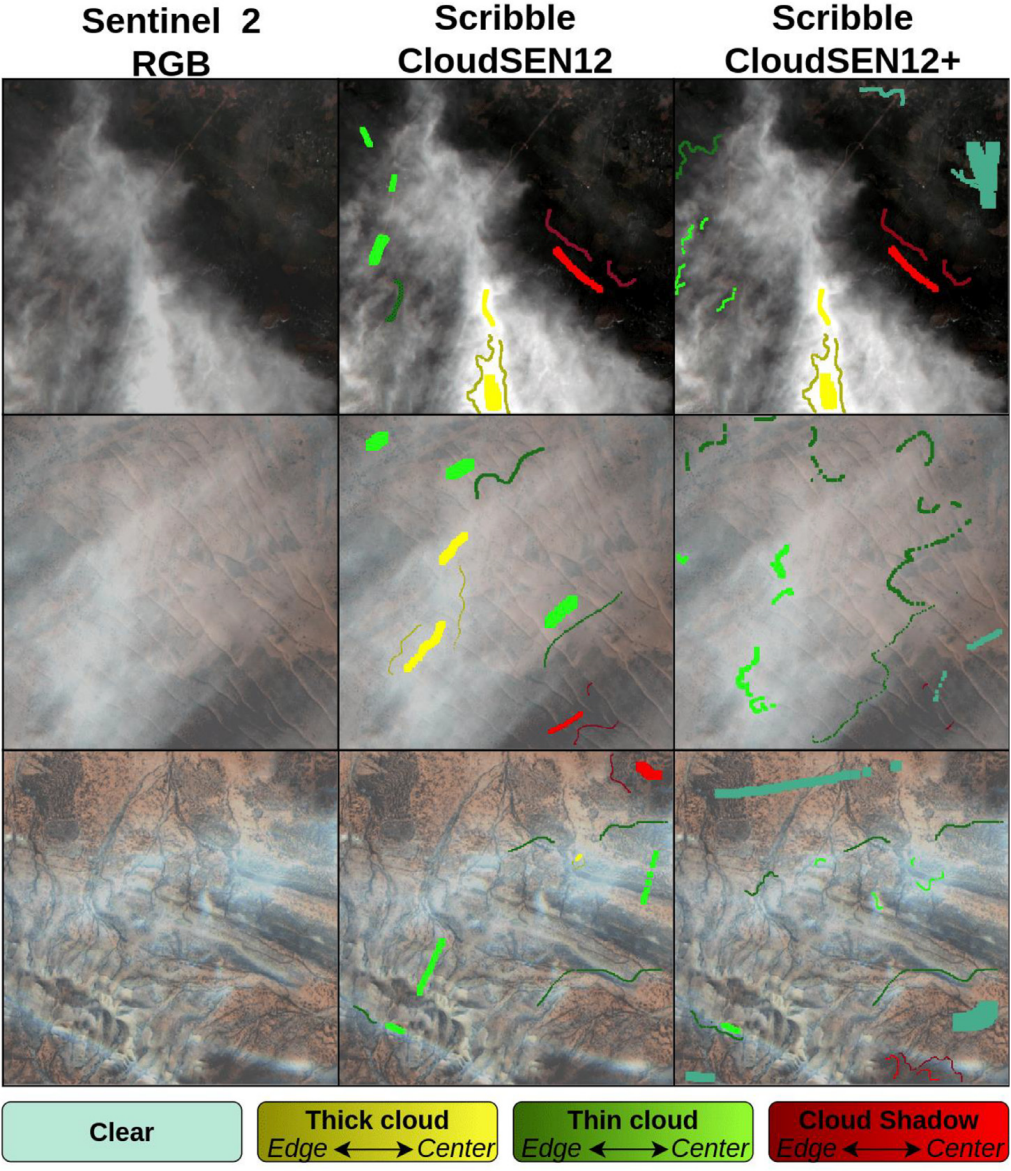
Correcting labels in the ‘scribble’ subset. These images originate from ROIs 1909, 3472, and 3474. The varying shades of yellow, green, and red represent the edges (darker) and center (lighter) of the annotations.

All the image patches flagged by *P_error_* undergo an extra visual inspection (see Fig. 6). This method flagged 17.12% of CloudSEN12+ annotations as potential errors (3,570 images). Upon visual inspection by the labeling team, 342 and 110 image patches from the high and scribble subsets were confirmed as real human errors. In Figs. 7 and 8, we present examples of human labeling before and after the review process.

## Limitations

As mentioned in the semi-automatic label quality section, the ground truth data relies on human interpretation, which is not infallible. While two rounds of validation have been performed on this dataset, some errors may remain, especially in complex areas with snow, faint cloud shadows, or thin clouds, where consensus was difficult to achieve. Nonetheless, these errors are expected to be minimal. After the second review, out of the 3,570 images examined (17.12 %), only 452 (12.6%) were found to have actual errors, with less than 1% being significant errors.

## Ethics Statement

This study does not involve any experimental procedures on human subjects or animals. The Sentinel program (primary data source) follows an open and free access policy for its data. Finally, this research complies with the terms of service provided by Google Earth Engine.

## CRediT Author Statement

**Cesar Aybar:** Conceptualization, Methodology, Software, Formal analysis, Writing- Original draft preparation. **Lesly Bautista:** Conceptualization, Methodology, Writing- Original draft preparation, Visualization, Validation. **David Montero:** Software, Formal analysis, Validation. **Julio Contreras:** Validation, Investigation, Data Curation. **Daryl Ayala:** Validation, Investigation, Data Curation. **Fernando Prudencio:** Validation, Visualization. **Jhomira Loja:** Validation, Investigation, Data Curation. **Luis Ysuhuaylas:** Validation, Investigation, Data Curation. **Fernando Herrera:** Validation, Investigation, Data Curation. **Karen Gonzales:** Validation, Investigation, Data Curation. **Jeanett Valladares:** Validation, Investigation, Data Curation. **Lucy A. Flores:** Validation, Investigation, Data Curation. **Evelin Mamani:** Validation, Investigation, Data Curation. **Maria Quiñonez:** Validation, Investigation, Data Curation. **Rai Fajardo:** Validation, Investigation, Data Curation. **Wendy Espinoza:** Validation, Investigation, Data Curation. **Antonio Limas:** Validation, Investigation, Data Curation. **Roy Yali:** Validation, Investigation, Data Curation. **Alejandro Alcántara:** Writing - Review & Editing, Funding acquisition. **Martin Leyva:** Writing - Review & Editing, Validation, Project administration. **Raul Loayza:** Writing - Review & Editing, Funding acquisition. **Bram Willems:** Writing - Review & Editing, Funding acquisition. **Gonzalo Mateo-García:** Conceptualization, Supervision, Writing - Review & Editing. **Luis Gómez-Chova:** Conceptualization, Supervision, Writing - Review & Editing, Funding acquisition.

## Data Availability

CloudSEN12+: The largest collection of expert-labeled pixels for cloud and cloud shadow detection in Sentinel-2 (Original data) (ScienceDataBank).
